# Metagenomic Insights Into the Role of Gut Microbes in the Defensive Ink “Tsunabi” of Physeteroid Whales

**DOI:** 10.1002/ece3.71910

**Published:** 2025-08-08

**Authors:** Hayate Takeuchi, Takashi Fritz Matsuishi, Takashi Hayakawa

**Affiliations:** ^1^ Division of Biosphere Science Graduate School of Environmental Science, Hokkaido University Sapporo Hokkaido Japan; ^2^ Global Center for Food, Land and Water Resources Faculty of Fisheries Sciences, Hokkaido University Hakodate Hokkaido Japan; ^3^ Section of Environmental Biology Faculty of Environmental Earth Science, Hokkaido University Sapporo Hokkaido Japan

**Keywords:** heavy metals, pigmentation, sperm whale, stranding, symbiosis, tryptophan metabolism

## Abstract

Whales of the superfamily Physeteroidea, which includes the genera *Physeter* and *Kogia*, exhibit a unique visual defense mechanism involving the release of dark reddish‐brown feces (locally called “tsunabi‐ink” in Japan) into the water to obscure themselves from predators and other threats. However, the mechanism underlying pigmentation remains unknown. Because physeteroids possess an enlarged distal colon that retains fecal material, a possible explanation is that symbiont microbial metabolism contributes to the feces pigmentation. To investigate this, we provided a shotgun metagenomic catalog of gut microbiomes from the intestinal tracts of eight cetacean species, including two physeteroids: a sperm whale ( *Physeter macrocephalus* ) and a pygmy sperm whale ( *Kogia breviceps* ). The colonic microbiome of physeteroids exhibited relatively high abundances of tryptophan metabolism genes, particularly indolepyruvate ferredoxin oxidoreductases (*iorA* and *iorB*), suggesting that physeteroids accumulate indole‐3‐pyruvate‐derived pigments in their colons. Furthermore, bacterial members of the phyla Bacillota and Bacteroidota were identified in the physeteroid colon as primary taxa conferring heavy‐metal resistance, which may be related to the primary predation of physeteroids on cephalopods, which bioaccumulate high levels of heavy metals. Prolonged fecal retention can expose gut microbes to chronic heavy‐metal stress and colonize them as heavy metal‐tolerant microbial communities, some of which may produce pigments to reduce their toxicity. Thus, we propose that tsunabi‐ink is a metabolic byproduct of shifts in the gut microbial community, influenced by the host's digestive physiology and foraging behavior through sustained ecological interactions with gut symbionts. Moreover, we believe that further empirical investigation would validate this hypothesis.

## Graphical abstract

Whales of the superfamily Physeteroidea exhibit a unique visual defense mechanism involving the release of dark reddish‐brown feces (locally called “tsunabi‐ink” in Japan) into the water. This study proposes that tsunabi‐ink is a metabolic byproduct of shifts in the gut microbial community, influenced by the host's digestive physiology and foraging behavior through sustained ecological interactions with gut symbionts.

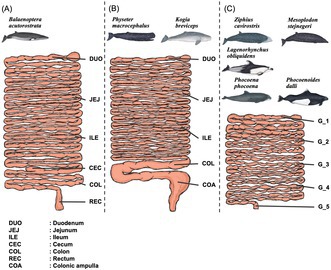

## 1 Introduction

The survival of prey animals depends on their ability to escape, deceive, or deter predators. One of the most striking defense strategies for prey animals is ink‐based evasion. These defense mechanisms are traditionally regarded as antipredator behaviors among invertebrates (Lang and Hescheler 1900; Derby 2014). Cephalopods, particularly coleoids, expel ink in various forms, including dark diffused clouds and pseudomorphs that mimic the shapes of other animals (Bush and Robison 2007). Melanin, the primary natural pigment in these inks, is synthesized within specialized ink sacs, giving the ink its characteristic black color (Palumbo 2003). Furthermore, sea hares (Anaspidea) possess ink glands in their mantle cavities and release purple ink containing aplysioviolin (Carefoot et al. 1999). Aplysioviolin, derived from the accessory photosynthetic pigment phycoerythrin in red algae through digestive processing, is subsequently synthesized in the ink glands (Nolen et al. 1995; Kamio et al. 2010). In both cases, ink production relies on a specialized storage sac or gland, along with enzymatic pathways that have evolved explicitly for this purpose.

**FIGURE 1:**
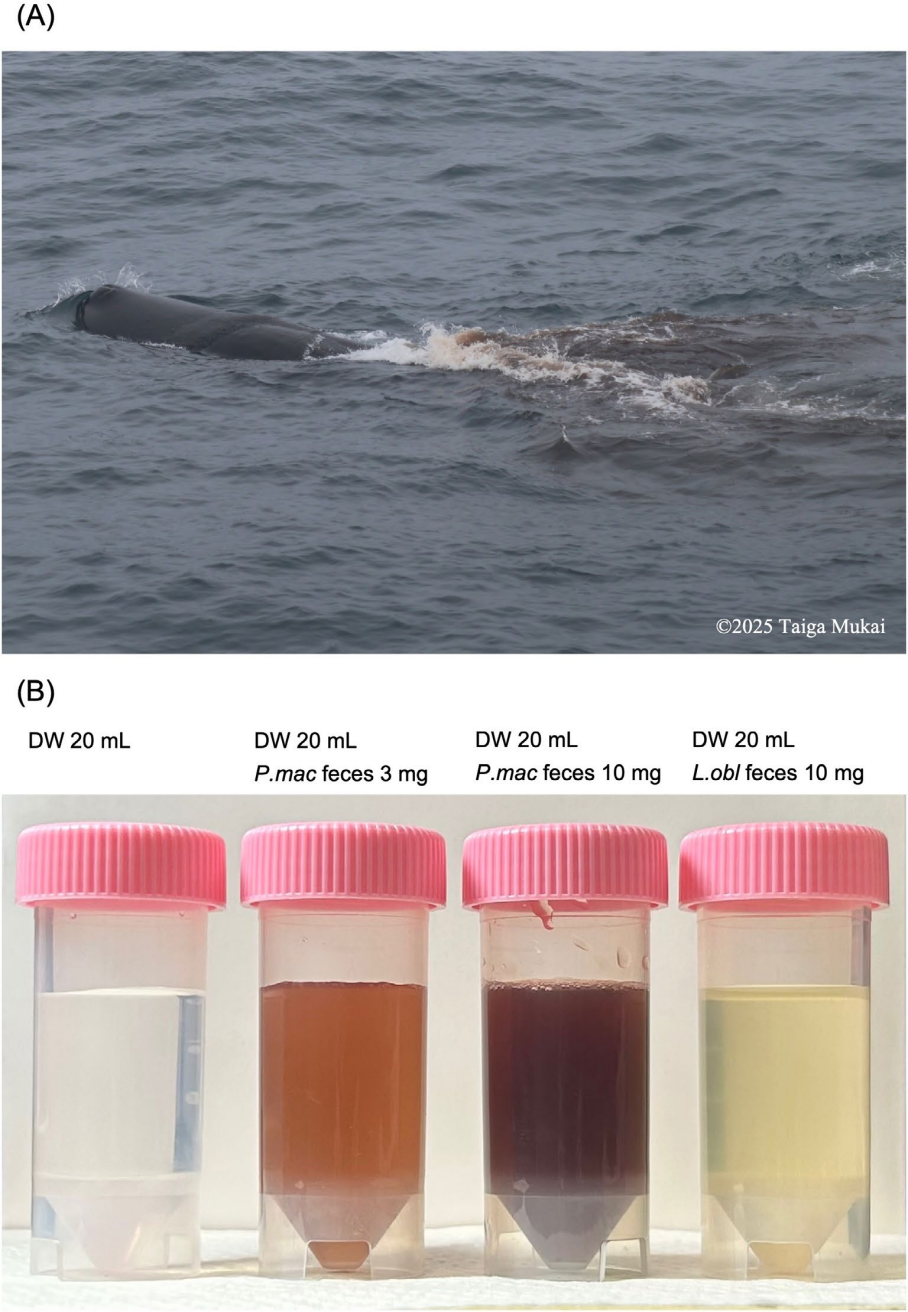
The appearance of tsunabi‐ink in *Physeter macrocephalus*. (A) A photograph of tsunabi‐ink released by *P. macrocephalus* , obtained at 42.26667° N, 152.12333° E. The whale's back and blowhole are visible to the left of the image, surfacing above the water. A plume of dark reddish‐brown feces (tsunabi‐ink) disperses into the seawater toward the right side of the frame. 2025 Taiga Mukai. (B) The color of the solutions prepared by dissolving whale feces in 20 mL of distilled water. From left to right, the tubes contain (1) 20 mL of distilled water only, (2) 3 mg of *P. macrocephalus* feces in 20 mL of distilled water, (3) 10 mg of *P. macrocephalus* feces in 20 mL of distilled water, and (4) 10 mg of *L. obliquidens* feces in 20 mL of distilled water. The solution containing 3 mg of *P. macrocephalus* feces showed that tsunabi‐ink exhibited a reddish‐brown hue. In the solution containing 10 mg of *P. macrocephalus* feces, the intense coloration indicates that tsunabi‐ink effectively serves as a smoke‐screen‐like visual deterrent against potential threats.

Despite being a hallmark of invertebrate defense strategies, “ink‐like” defense mechanisms are rare among vertebrates. Notably, such behavior has been documented in three species of the superfamily Physeteroidea, namely, the sperm whale ( *Physeter macrocephalus* ), the pygmy sperm whale ( *Kogia breviceps* ), and the dwarf sperm whale ( *K. sima* ), thereby representing an intriguing case of convergent evolution with invertebrates in terms of antipredator adaptations (Figure 1A,B; Yamada 1954; Scott and Cordaro 1987; Merkens et al. 2018; Levesque et al. 2023). The term “tsunabi” coined by Japanese seafarers to describe pygmy sperm whales (Yamada 1954). Tsunabi derives its name from the behavior exhibited by pygmy sperm whales, whereby they expel dark reddish‐brown evacuations that bear a resemblance to bleeding, while simultaneously disappearing into the water in response to sudden attacks from potential predators and other threats (Yamada 1954). Chuhei Mizutani reasonably asserted that this name was associated with the traditional Japanese firework tsunabi, which propelled a fiery trail along a rope line (Yamada 1954).

The chemical composition and physiological mechanisms underlying tsunabi remain virtually unexplored. Following its rescue and subsequent care in a Japanese aquarium, a live‐stranded pygmy sperm whale exhibited the distinctive defensive inking behavior (Soichi et al. 2009). When housed with other species, such as bottlenose dolphins and Risso's dolphins, the pygmy sperm whale released large quantities of reddish‐brown fecal material and used the resulting ink cloud as a visual shield against pursuing individuals. Soichi et al. (2009) termed this specific behavior the “tsunabi reaction,” naming it after the pygmy sperm whale's alias tsunabi. Interestingly, both *Kogia* and *Physeter* have been documented as prey for apex predators. *Kogia* species are known to be consumed by large sharks such as the white shark ( *Carcharodon carcharias* ) (Long and Jones 1996), and killer whales ( *Orcinus orca* ) have been observed preying upon *Kogia* spp. in the Bahamas (Dunn and Claridge 2014). In one notable case, a pod of approximately 35 killer whales attacked a group of nine *P. macrocephalus* , resulting in the death and consumption of one individual and serious injuries to the others (Pitman et al. 2001). These observations highlight that physeteroid species may face significant predation risks, particularly from killer whales and large sharks. Such ecological pressures may provide a plausible context in which the stress‐induced release of ink‐like fecal material could serve a protective or evasive function by obscuring the body or distracting predators. Despite its intriguing nature, the tsunabi reaction has rarely been documented, making it one of the least understood defensive behaviors among marine mammals.

In contrast to cephalopods and sea hares, which possess specialized ink sacs or glands, no dedicated organ for the storage of ink has been reported in physeteroids. Rather, the tsunabi‐ink is stored within the digestive tract, specifically in the uniquely enlarged distal colon, subsequently referred to as the colonic ampulla. This anatomical feature, which is absent in other cetaceans, is characterized by the retention and accumulation of dark reddish‐brown feces, indicating that the coloration of tsunabi may be a byproduct of digestive processes. Given that the ink‐like substance accumulates as intestinal contents, one possible explanation points to microbial metabolism within the colonic ampulla, along with host endogenous digestive processes, contributing to tsunabi pigmentation. If particular gut microbial metabolic processes influence tsunabi pigmentation, they may interact with dietary compounds, host endogenous secretions, or trace elements retained during fecal storage.

This study provided a catalog of shotgun metagenomes of gut microorganisms across various intestinal sites in eight wild‐derived cetacean species, including *P. macrocephalus* and *K. breviceps* , with the aim of exploring the genetic potential of gut microorganisms for pigment production. These data provide an opportunity to address the following two fundamental questions: (1) Do symbiotic gut microorganisms have the potential to contribute to pigment biosynthesis through metabolic and genetic pathways? (2) Are specific groups of gut bacteria consistently involved in tsunabi‐ink production? This establishes a foundation for understanding how gut microbial metabolism integrates into the broader framework of feeding ecology and digestive adaptations of physeteroids in tsunabi pigmentation.

## 2 Materials and Methods

### 2.1 Ethics Approval

We collected the samples with the Stranding Network Hokkaido (SNH) (Stranding Network Hokkaido 2024a, 2024b, 2024c, 2024d, 2024e, 2024f, 2024g, 2024h, 2024i). The process complied with Japan's relevant laws and regulations, and SNH provided the samples unconditionally for this study.

### 2.2 Sample Collection

The SNH conducted necropsies on nine recently deceased individuals representing eight species of cetaceans, procuring samples of intestinal contents (Table 1; Stranding Network Hokkaido 2024a, 2024b, 2024c, 2024d, 2024e, 2024f, 2024g, 2024h, 2024i). All samples were collected between 2021 and 2023. The first two digits of each SNH sample ID indicate the year of collection (e.g., SNH22047 was collected in 2022). The samples collected included those from the ink‐producing species *P. macrocephalus* and *K. breviceps* , in addition to other cetaceans: the minke whale ( *Balaenoptera acutorostrata* ), Cuvier's beaked whale ( *Ziphius cavirostris* ), Stejneger's beaked whale ( *Mesoplodon stejnegeri* ), the Pacific white‐sided dolphin ( *Lagenorhynchus obliquidens* , recently reclassified as *Aethalodelphis obliquidens*), the harbor porpoise ( *Phocoena phocoena* ), and Dall's porpoise ( *Phocoenoides dalli* ).

**TABLE 1:** Specimen IDs assigned by the Stranding Network Hokkaido (SNH) and reference genomes for host read removal.

Species		SNH ID	Citation of specimen	Accession number of reference genome	Citation of reference genome
Minke whale	*Balaenoptera acutorostrata*	SNH22047	Stranding Network Hokkaido (2024a)	GCF_000493695.1	Yim et al. (2014)
Sperm whale	*Physeter macrocephalus*	SNH21048	Stranding Network Hokkaido (2024b)	GCF_002837175.3	Fan et al. (2019)
Pygmy sperm whale	*Kogia breviceps*	SNH23034	Stranding Network Hokkaido (2024c)	CNS0152179	Yuan et al. (2021)
Cuvier's beaked whale	*Ziphius cavirostris*	SNH22037	Stranding Network Hokkaido (2024d)	GCA_004364475.1	Zoonomia Consortium (2020)
Stejneger's beaked whale	*Mesoplodon stejnegeri*	SNH21009	Stranding Network Hokkaido (2024e)	GCA_004027085.1	Zoonomia Consortium (2020)
Pacific white‐sided dolphin	*Lagenorhynchus obliquidens*	SNH22023	Stranding Network Hokkaido (2024f)	CNS0152182	Yuan et al. (2021)
Harbor porpoise	*Phocoena phocoena*	SNH21015	Stranding Network Hokkaido (2024g)	GCA_003071005.2	Autenrieth et al. (2018)
Harbor porpoise	*Phocoena phocoena*	SNH22020	Stranding Network Hokkaido (2024h)	GCA_003071005.2	Autenrieth et al. (2018)
Dall's porpoise	*Phocoenoides dalli*	SNH23008	Stranding Network Hokkaido (2024i)	GCA_003071005.2	Autenrieth et al. (2018)

The specimen identification numbers assigned by the SNH are presented in Table 1. The sperm whale and Cuvier's beaked whale were alive at the time of stranding; however, they were subsequently confirmed as deceased. The Pacific white‐sided dolphin and harbor porpoise were accidentally caught in a set‐net fishery in Usujiri, Hakodate, Japan, and their status was later confirmed as deceased. The pygmy sperm whale and Stejneger's beaked whale were found deceased upon discovery, although their carcasses exhibited an exceptionally fresh condition. Additionally, the minke whale and Dall's porpoise were also identified as deceased at the time of stranding, although their carcasses were less fresh than those of the other specimens.

**FIGURE 2:**
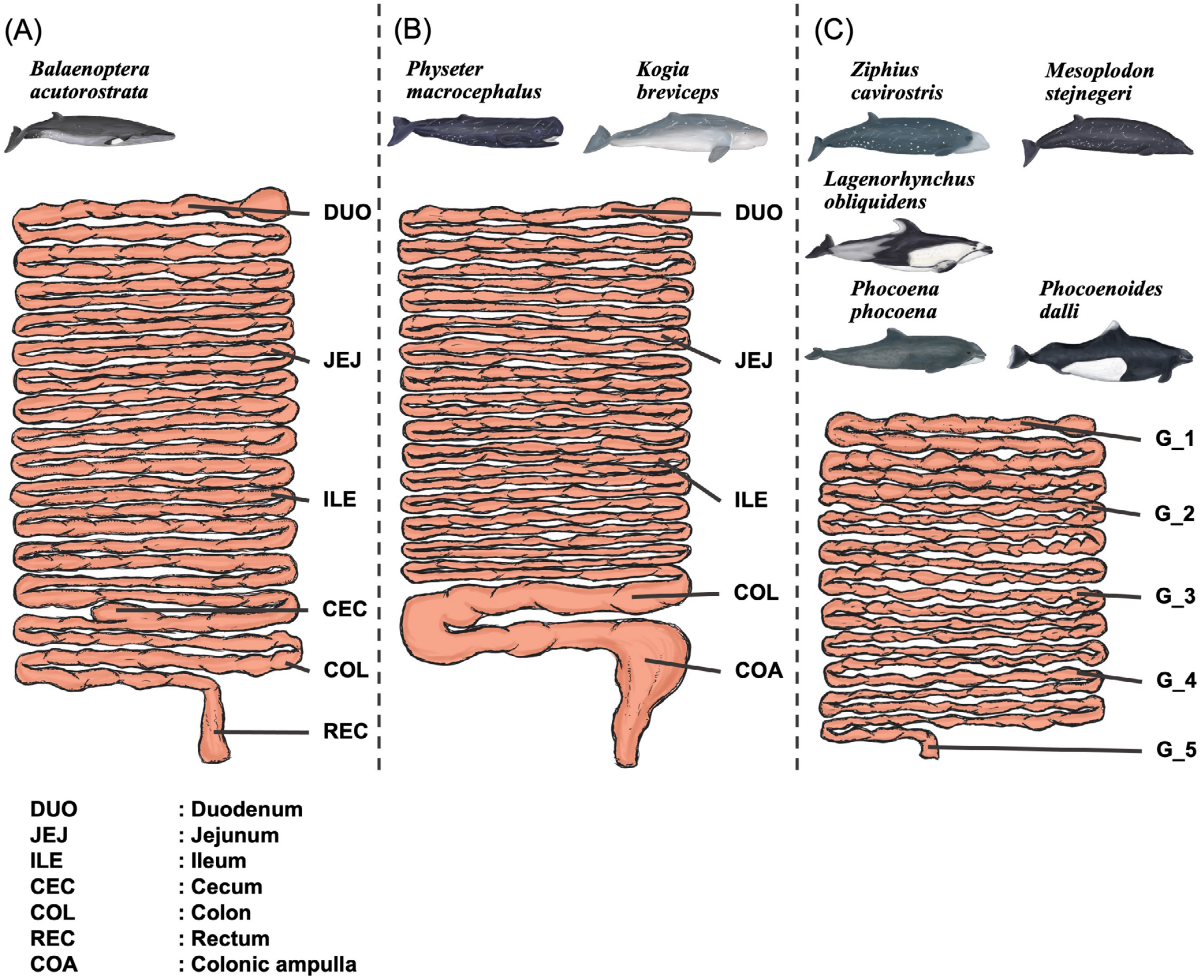
A diagram of the sampling locations. (A) *Balaenoptera acutorostrata* possesses a cecum. The intestinal contents were sampled from the duodenum, jejunum, and ileum (proximal to the cecum) and the colon and rectum (distal to the cecum). (B) In *Physeter macrocephalus* and *Kogia breviceps*, the diameter of the distal intestine significantly increases. Samples were collected from the duodenum, jejunum, and ileum (proximal to the enlarged region) and from the colon and colonic ampulla within the enlarged distal section. (C) In the remaining species, five equidistant sampling points (G_1–G_5) were established along the intestinal tract from the gastric to the anal end, and the contents were collected accordingly. Each symbol corresponds to a distinct region: Duodenum (DUO), jejunum (JEJ), ileum (ILE), cecum (CEC), colon (COL), rectum (REC), and colonic ampulla (COA).

The intestinal contents were collected from five or six distinct sites along the intestinal tract of each subject. In summary, we obtained six samples from the minke whale, which possesses a cecum, utilizing the cecum as a reference point: the duodenum, jejunum, ileum, cecum, colon, and rectum contents (Figure 2A). In the case of the sperm whale and pygmy sperm whale, sampling was conducted based on variations in intestinal diameter, with samples acquired from the duodenum, jejunum, ileum, colon, and colonic ampulla (Figure 2B). The remaining five species had a relatively uniform intestinal structure, thereby making it difficult to distinguish specific sections (Figure 2C). Therefore, samples were obtained from five equidistant sites along the tract, ranging from the proximal (gastric) end to the distal (anal) end (Figure 2C).

The collected samples were promptly frozen at −20°C, transferred to a −80°C freezer as soon as possible, and stored at −80°C until total DNA extraction. In total, 63 samples from eight cetacean species were subjected to shotgun metagenomic sequencing. This includes several technical replicates generated from the same individual when the initial sequencing depth was insufficient. All samples and replicates are listed in Table 2. The number and type of samples per species or individual reflect the opportunistic nature of cetacean necropsy and sample availability, rather than a balanced experimental design.

**TABLE 2:** Shotgun metagenomic statistics and assembly metrics.

Species	ID	Raw reads	Host removal reads		%Q30	GC content	Total assembled bases	Contig N50
		Total bases	Total reads	Total bases				
Minke whale	DUO_a^a^, ^i^	5.190145 G	12,560,600	1,706,519,350	94.069340%	36.027699%	51,138,946	5246
	DUO_b^j^	6.724656 G	13,722,176	1,898,427,449	97.371246%	35.572227%	38,477,792	7007
	JEJ_a^b^, ^i^	8.992993 G	17,397,394	2,522,179,100	94.458584%	34.047992%	52,461,514	3627
	JEJ_b^j^	8.427803 G	11,614,598	1,321,721,504	97.723144%	34.417856%	26,783,491	9038
	ILE_a^c^, ^i^	9.915424 G	6,975,682	1,013,250,542	94.084378%	37.394836%	32,180,747	6361
	ILE_b^j^	18.281255 G	6,624,160	729,295,588	97.410436%	38.010437%	17,255,621	3249
	CEC_a^d^, ^i^	5.787769 G	9,314,926	1,135,420,735	92.331975%	41.761301%	97,097,171	708
	CEC_b^j^	7.592211 G	12,263,950	1,051,539,163	97.631648%	40.524296%	42,499,868	537
	COL_a^e^, ^i^	12.331224 G	14,625,234	1,576,934,488	95.347550%	40.333203%	113,997,451	724
	COL_b^j^	16.169756 G	19,928,292	1,940,230,554	97.636779%	40.890115%	88,041,138	624
	REC_a^f^, ^i^	4.467009 G	3,878,384	361,941,871	95.550871%	40.774891%	21,166,752	467
	REC_b^j^	15.910852 G	14,336,126	1,377,667,678	97.733345%	41.490057%	68,150,001	592
	ALL^g^	—	—	—	—	—	291,540,209	1549
Sperm whale	DUO_a^i^	5.216858 G	8,779,384	763,437,956	95.064064%	37.114943%	9,357,531	6909
	DUO_b^j^	6.208277 G	7,860,908	853,449,060	98.048416%	36.175520%	9,910,558	12,834
	JEJ^j^	19.711453 G	7,583,968	888,584,832	97.399338%	46.066696%	8,730,504	9733
	ILE_a^i^	1.746701 G	720,674	97,572,330	93.267841%	42.887272%	9,416,755	21,547
	ILE_b^j^	12.359472 G	5,079,080	435,787,880	97.589932%	39.763754%	5,508,838	1440
	COL_a^i^	9.915379 G	28,539,846	3,739,051,897	93.580187%	46.420130%	171,789,810	1257
	COL_b^j^	8.214224 G	19,375,984	2,215,192,832	97.775680%	44.520532%	78,038,849	720
	COA_a^h^ ^,^, ^i^	8.856102 G	25,417,386	3,412,884,064	93.461344%	45.131008%	135,035,888	1088
	COA_b^j^	9.439445 G	25,847,252	3,313,729,248	97.356528%	44.088857%	93,915,668	1058
	ALL	—	—	—	—	—	303,947,622	1884
Pygmy sperm whale	DUO^j^	9.849863 G	462,598	59,810,500	97.351600%	39.620052%	4,518,496	519
	JEJ^j^	8.305067 G	15,798,192	1,707,946,390	97.773817%	40.280347%	7,823,323	16,414
	ILE^j^	7.911075 G	7,399,024	819,304,952	97.596650%	38.529392%	8,507,703	16,933
	COL^j^	6.533490 G	12,154,258	1,446,147,154	97.539787%	44.417916%	84,708,615	980
	COA^j^	8.954376 G	16,937,690	1,739,913,301	97.705901%	43.075025%	74,779,769	652
	ALL	—	—	—	—	—	173,738,984	905
Cuvier's beaked whale	G_1_a^i^	6.689217 G	321,188	44,394,087	93.795214%	46.667660%	6,848,062	590
	G_1_b^j^	17.934648 G	571,308	67,335,711	97.358865%	44.725600%	5,062,825	540
	G_2_a^i^	335.131514 M	57,250	8,319,223	93.397458%	43.830121%	1,068,369	438
	G_2_b^j^	20.585256 G	368,156	46,319,729	97.142176%	41.844471%	3,013,897	491
	G_3_a^i^	102.284078 M	29,448	4,269,905	93.612054%	42.416915%	446,306	427
	G_3_b	21.021226 G	401,092	46,390,082	97.447160%	41.830435%	1,993,461	678
	G_4_a^i^	100.252222 M	38,542	4,433,137	93.979830%	42.463804%	34,643	365
	G_4_b^j^	15.142844 G	165,658	20,577,551	97.472761%	41.022958%	582,375	380
	G_5_a^i^	2.273013 G	571,530	77,798,369	93.532392%	42.139691%	10,817,922	780
	G_5_b^j^	14.387059 G	5,307,816	678,739,800	97.398619%	39.340643%	26,819,897	1292
	ALL	—	—	—	—	—	47,293,807	976
Stejneger's beaked whale	G_1_a^i^	7.444453 G	727,536	77,631,250	95.248323%	42.551315%	8,048,577	1609
	G_1_b^j^	17.710236 G	1,989,052	209,992,212	97.266132%	40.288271%	8,668,034	2344
	G_2_a^i^	6.921018 G	10,014,848	1,297,162,924	94.608621%	32.538769%	28,215,391	3510
	G_2_b^j^	7.653894 G	6,720,484	757,394,488	97.823100%	31.572302%	17,494,195	3085
	G_3_a^i^	7.397987 G	713,772	88,989,286	94.042120%	44.704188%	8,945,745	1939
	G_3_b	10.167209 G	878,978	91,794,087	97.455601%	43.198994%	5,866,773	940
	G_4_a^i^	5.888801 G	9,593,192	1,070,746,024	95.088257%	36.531129%	21,072,708	4310
	G_4_b^j^	7.297450 G	10,918,730	1,119,663,367	97.557709%	36.107303%	16,810,484	3490
	G_5_a^i^	7.392384 G	7,930,556	826,355,097	95.228926%	33.838883%	29,540,544	11,217
	G_5_b^j^	9.951532 G	14,425,138	1,982,474,168	97.371161%	33.561739%	33,743,526	43,069
	ALL	—	—	—	—	—	51,346,442	20,747
Pacific white‐sided dolphin	G_1_a^i^	5.469702 G	786,308	108,337,828	93.808204%	42.860852%	5,439,075	153,632
	G_1_b^j^	8.342381 G	4,329,606	440,256,899	97.522941%	38.952575%	5,585,529	69,953
	G_2^j^	21.107130 G	1,337,692	149,441,245	97.393355%	39.876615%	9,678,816	1042
	G_3^j^	9.154753 G	4,458,788	481,732,854	97.612875%	38.967208%	16,992,875	4720
	G_4^j^	10.509492 G	179,496	20,162,944	97.680845%	38.781183%	236,517	418
	G_5^j^	20.698105 G	16,904,610	2,155,892,882	97.532067%	35.938839%	55,749,962	3863
	ALL	—	—	—	—	—	58,974,974	4682
Horbor porpoise	G_1_a^i^, ^k^	5.998205 G	325,540	35,273,676	94.695908%	45.479270%	2,126,858	1324
	G_1_b^j^	22.003802 G	79,088	9,305,167	97.018316%	38.818288%	436,070	460
	G_2^j^	9.921232 G	153,568	18,069,499	97.227955%	39.432829%	883,221	482
	G_3^j^	8.697475 G	68,262	8,519,154	97.107466%	39.131066%	416,344	464
	G_4^j^	22.244908 G	150,082	19,574,482	97.391622%	39.567554%	1,104,167	478
	G_5^j^	18.561954 G	1,025,302	114,957,058	97.195724%	44.099338%	6,839,577	594
	ALL	—	—	—	—	—	12,670,383	626
Dall's porpoise	G_1^j^	6.295230 G	485,624	58,820,731	97.306835%	39.705772%	2,737,385	7457
	G_2^j^	12.152482 G	189,982	21,080,042	97.236093%	40.964712%	526,660	430
	G_3^j^	9.074009 G	83,956	8,429,283	97.512401%	39.321467%	157,313	467
	G_4^j^	19.390350 G	347,244	40,668,609	97.280387%	40.160979%	3,919,597	676
	G_5^j^	8.263361 G	5,179,288	688,711,243	97.387050%	42.794030%	22,382,203	5224
	ALL	—	—	—	—	—	27,759,740	4383

^a^ DUO: duodenum.

^b^ JEJ: jejunum.

^c^ ILE: ileum.

^d^ CEC: cecum.

^e^ COL: colon.

^f^ REC: rectum.

^g^ ALL: the merged sample per species.

^h^ COA: colonic ampulla.

^i^ Illumina HiSeq X platform.

^j^ NovaSeq X Plus platform.

^k^ Sample is from a harbor porpoise with the specimen ID SNH21015.

Note that this study did not include sufficient biological and technical replicates because necropsy‐based fresh sampling of wild whales is highly opportunistic and limited. SNH aims to distribute samples and provide equal opportunities to researchers who do not necessarily conduct genetic research studies; therefore, we sought to minimize our genetic sampling efforts. Alternatively, to confirm reproducibility, we could analyze two physeteroid species or other species as groups of tsunabi‐producing or tsunabi‐non‐producing species.

### 2.3 DNA Extraction, Library Preparation, and Metagenomics Sequencing

Total DNA was extracted using a QIAamp Fast DNA Stool Mini Kit (QIAGEN, N.V., Hilden, Germany). Subsequently, the concentration and purity of the total DNA solution were measured using a NanoDrop 2000 (Thermo Fisher Scientific, Waltham, MA, USA) and an Invitrogen Qubit 2.0 Fluorometer (Thermo Fisher Scientific). The quality of the DNA extract was assessed using a 5% agarose gel.

A metagenomic library with an insert size of 300 bp was constructed from high‐quality DNA extracted from each sample using the NEBNext Ultra II DNA Library Preparation (New England Biolabs, Ipswich, MA, USA), NEBNext Multiplex Oligos for Illumina (Dual Index Primers Set) (New England Biolabs), and AMPure XP Beads (Beckman Coulter Inc., Brea, CA, USA) following the manufacturer's manual instructions. The Agilent 2200 TapeStation system (Agilent Technologies, Santa Clara, USA) was used to determine the fragment size distribution.

Metagenomic sequencing was conducted utilizing 150‐bp paired‐end reads on the Illumina HiSeq X or NovaSeq X Plus platform (Illumina, San Diego, CA, USA) (see Table 2 for sequencing details). The sequencing generated 647 Gb of data, comprising 4228 million reads from 63 samples.

### 2.4 Quality Control, Metagenome Assembly, and Metagenome Binning

Quality control and preprocessing of shotgun metagenome data were performed using fastp v0.23.4 (Chen et al. 2018). This process involved filtering out low‐quality reads characterized by a mean Phred quality score below 20, read adapters, reads shorter than 15 bases, and sequences exhibiting low complexity of consecutive bases in the poly‐G region sequences. The filtered reads were mapped to the respective host genome sequences using the BWA‐MEM algorithm from the Burrows‐Wheeler Aligner v0.7.18‐r1243‐dirty and processed with samtools v1.21 to remove host‐derived reads (Li and Durbin 2009; Li 2013; Danecek et al. 2021). The accession numbers and source papers for the host cetacean genome sequences used are listed in Table 1. If a reference genome for the target species was unavailable, the genome of a closely related species was used.

After host reads were removed, all samples were assembled individually using MEGAHIT v1.2.9 (Li et al. 2015) with default parameters optimized for metagenome assembly (*k*‐mer sizes of 21, 41, 61, 81, and 99). In addition, species‐level co‐assemblies were generated by pooling the filtered reads from all available samples within each species. These co‐assemblies were used to enhance contig continuity and support comparative analysis at the species level. As a result, the assembly yielded 2,688,870,863 bp of contigs with an average contig N50 of 7064 bp (Table 2).

Contigs longer than 1000 bp were systematically categorized into metagenome‐assembled genomes (MAGs) based on their sequence composition and coverage depth, using MaxBin v2.2.7 (Wu et al. 2016) and CONCOCT v1.1.0 (Alneberg et al. 2014). Additionally, contigs longer than 1500 bp were binned into MAGs employing MetaBAT 2 v2.17 (Kang et al. 2019). These thresholds of contig length used in binning represent the lowest contig length recommendations for each tool that are still considered acceptable for binning and were chosen to maximize the inclusion of fragmented but informative contigs. It should be acknowledged that using the minimal acceptable thresholds may increase the risk of incomplete or mis‐binned MAGs and introduce challenges in cross‐tool comparability. To address this, we applied all tools consistently across datasets and subsequently evaluated and refined the resulting MAGs using the DAS tool, dRep, and CheckM to ensure quality and comparability.

Subsequently, DAS Tool v1.1.1 was utilized to integrate the MAGs generated using various binning methodologies (Sieber et al. 2018). The resultant MAGs were then refined utilizing dRep v3.5.0 at a 95% ANI cutoff (Olm et al. 2017), yielding 167 nonredundant MAGs. The completeness and contamination of all MAGs were assessed using CheckM v1.0.11 with its lineage_wf workflow (Parks et al. 2015). Bins exhibiting a completeness of over 50% and contamination below 10% were designated as medium‐quality MAGs (*n* = 110). In comparison, those with a completeness above 90% and contamination below 5% were categorized as high‐quality MAGs (*n* = 57). The genome sizes of the MAGs were adjusted for completeness and contamination using a previously reported formula

$$\hat{G}=G/\hat{C}-G\cdot \hat{T}$$

where $\hat{G}$ represents the estimated genome size of the MAG, G is the observed genome size, $\hat{C}$ is the estimated completeness expressed as a ratio, and $\hat{T}$ is the estimated contamination expressed as a ratio (Nayfach et al. 2019).

### 2.5 Taxonomic Assignment and Functional Annotation

Based on the host‐depleted reads, taxonomic compositional annotation was performed for each sample using FOCUS v1.8 (Silva et al. 2014). FOCUS employs non‐negative least squares to determine the optimal set of organismal abundances by aligning the *k*‐mer composition of the metagenome with a reference database. The composition of the gut microbiome at the phylum level was visualized using the R package “ggplot2” (Wickham 2016; R Core Team 2024). All medium‐ and high‐quality MAGs underwent taxonomic annotation employing GTDB‐Tk v0.1.6, based on the GTDB Release R207_v2 (Hyatt et al. 2010; Matsen 2010; Eddy 2011; Jain et al. 2018; Chaumeil et al. 2019, 2022).

Open reading frames (ORFs) were predicted from the contigs assembled by MEGAHIT using Prodigal v2.6.3 with the parameter “‐p meta” (Hyatt et al. 2010). All ORFs obtained for each species were clustered at a 95% global sequence identity threshold to eliminate redundant sequences using CD‐HIT v4.8.1 (Li and Godzik 2006; Fu et al. 2012). These procedures identified 1,104,196 complete ORFs with an average length of 277 bp. Accumulation curves illustrating the number of subsampled ORF sequences were generated for each species, predicated upon a 95% local sequence identity clustering criterion employing DIAMOND DeepClust within DIAMOND v2.1.9.163 (Buchfink et al. 2021, 2023). Similarly, ORFs from all species were pooled, and accumulation curves were produced utilizing clustering thresholds of 50%, 75%, and 95% local sequence identity.

The nonredundant ORFs for each species underwent taxonomic and functional annotation using eggNOG‐Mapper v2.1.12 against the DIAMOND database (Huerta‐Cepas et al. 2019; Buchfink et al. 2021; Cantalapiedra et al. 2021). Default parameters were used, including an *E*‐value threshold of 1e−5. For taxonomic annotation, the lowest possible taxonomic level was inferred based on the eggNOG orthologous groups assigned by the DIAMOND search. The functional annotation comprised KEGG pathways/modules/orthologs (Kanehisa et al. 2017), Gene Ontology terms (Gene Ontology Consortium 2019), carbohydrate‐active enzymes (CAZy) (Lombard et al. 2014), enzyme commission numbers, BiGG reactions (Norsigian et al. 2020), PFAM protein domain (Mistry et al. 2021), and Clusters of Orthologous Groups functional categories (Tatusov et al. 2000). In order to identify heavy‐metal resistance genes pertaining to copper, cadmium, and iron within the query ORFs, a similarity search was conducted against the BacMet2 Predicted Resistance Genes Database v2.0 utilizing DIAMOND v2.1.9.163 (Pal et al. 2014; Buchfink et al. 2021). The search was conducted with an *E*‐value threshold of 1e−5.

In order to estimate the relative abundance of genes, quality‐filtered sequences from each sample were lightweight‐mapped to ORFs using Salmon v1.10.3 (Patro et al. 2017). Salmon calculated the relative abundance of ORFs in transcripts per million (TPM), which were normalized according to the sample's gene length and the sequencing depth. It is important to emphasize that TPM values hold significant relevance solely for comparisons within the same ORF dataset, set, i.e., either among different intestinal regions of the same species or across the same intestinal locations of that species. Nonetheless, to accommodate interspecies variability in the number of annotated genes, the TPM values were standardized in relation to the quantity of annotated genes present in the *P. macrocephalus* samples. Specifically, the TPM values for each gene in the other species were adjusted by multiplying them by the ratio of the number of annotated ORFs in that species to the number of annotated ORFs in *P. macrocephalus* . This adjustment enabled the visualization of relative gene abundances across species on the same TPM scale; however, it remains an arbitrary normalization with no inherent biological significance.

To identify bacterial taxa potentially constituting a functional core microbiome associated with tryptophan metabolism and heavy metal resistance, we annotated all predicted ORFs using eggNOG‐mapper (for KEGG pathway ko00380) and the BacMet2 predicted resistance gene database (for cadmium, copper, and iron resistance). Taxonomic assignments were made at the family level based on eggNOG‐mapper annotations. For *P. macrocephalus* and *K. breviceps* , we calculated the average TPM values of function‐associated ORFs within colonic regions (colon and colonic ampulla). An alluvial diagram was produced utilizing the R packages “ggalluvial,” “alluvial,” and “ggplot2” to illustrate the relationship between gene functions and the relative abundance of microbial taxa that exhibit these functions. (Bojanowski and Edwards 2016; Wickham 2016; Brunson and Read 2020; R Core Team 2024).

### 2.6 Phylogenetic Tree Inference

All medium‐ and high‐quality MAGs underwent approximate maximum likelihood tree inference using the GTDB‐Tk v0.1.6 “gtdbtk infer” command, based on the WAG protein substitution model (Whelan and Goldman 2001; Hyatt et al. 2010; Matsen 2010; Price et al. 2010; Eddy 2011; Jain et al. 2018; Chaumeil et al. 2019, 2022). The phylogenetic tree inference for ORFs annotated with specific genes was conducted using the following procedure: First, multiple sequence alignment was conducted using MAFFT v7.525 with the default parameters (Katoh and Standley 2013). Subsequently, automatic trimming was applied using trimAl v0.1.6 with the “‐gappyout” option (Capella‐Gutiérrez et al. 2009). Finally, the maximum likelihood phylogenetic trees were inferred using IQ‐TREE 2 v2.3.4, in which the substitution model was automatically selected with the “‐m MFP + MERGE” command, and bootstrap analysis was conducted with 10,000 replicates (Chernomor et al. 2016; Kalyaanamoorthy et al. 2017; Hoang et al. 2018; Minh et al. 2020).

### 2.7 Alpha and Beta Diversity Analysis

To assess microbial diversity across intestinal regions and species, we computed both α‐diversity and β‐diversity metrics based on the taxonomic profiles generated by Focus. For α‐diversity, we calculated observed richness and Pielou's evenness at the order level (Pielou 1966). For β‐diversity, we calculated Aitchison distance after centered log‐ratio (CLR) transformation of the relative abundance data. Ordination was performed using non‐metric multidimensional scaling (NMDS) to visualize differences in microbial composition across samples (Aitchison et al. 2000). These indices were computed using an in‐house Python script based on the count table of classified taxa.

## 3 Results

### 3.1 Taxonomic Composition of the Microbial Communities

**FIGURE 3:**
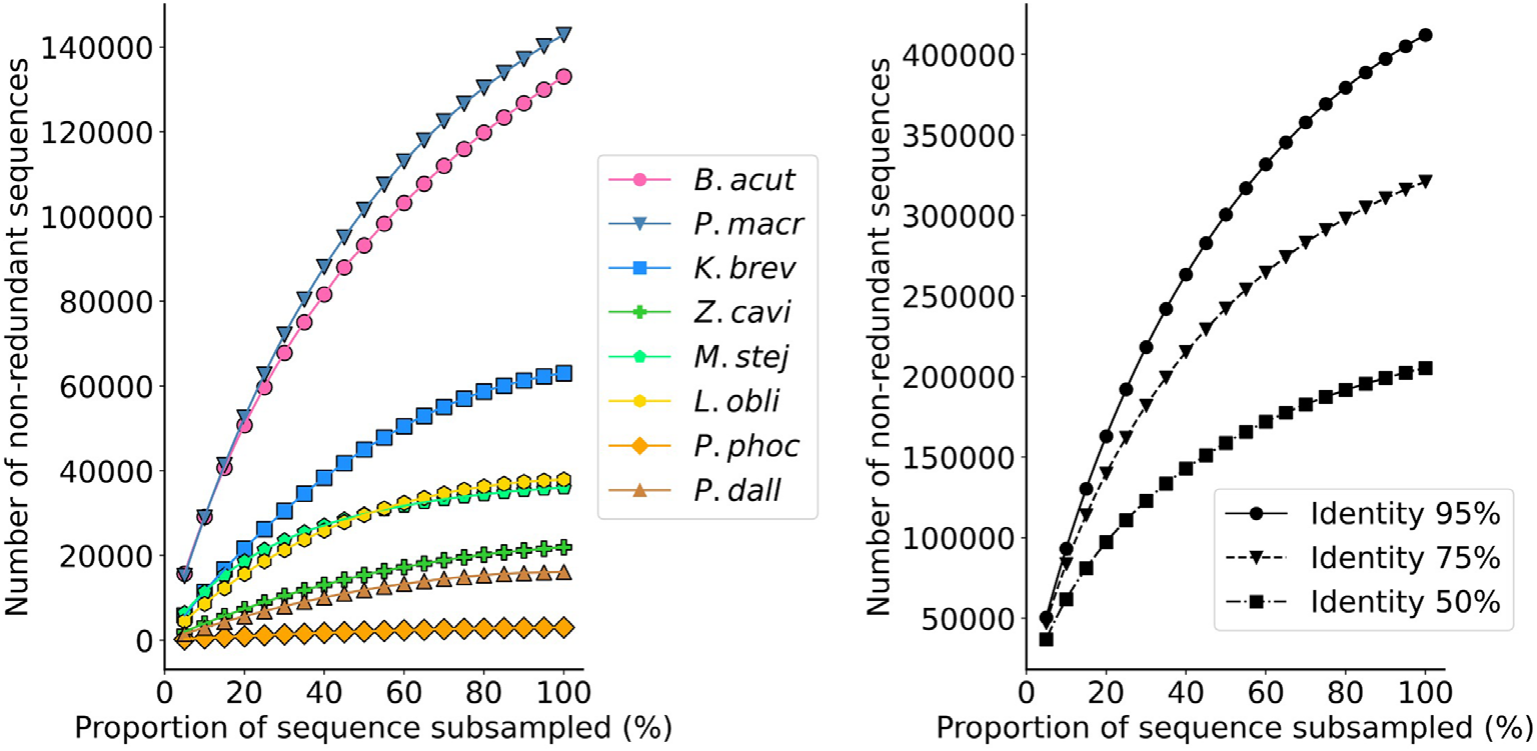
Accumulation curves of metagenomic open reading frames (ORFs). (Left) Accumulation curves of ORFs obtained from each cetacean species at a 95% local sequence identity clustering threshold. (Right) Accumulation curves of ORFs obtained from all cetacean species at 95%, 75%, and 50% local sequence identity clustering thresholds. *B. acut*: Minke whale, *P. macr*: Sperm whale, *K. brev*: Pygmy sperm whale, *Z. cavi*: Cuvier's beaked whale, *M. stej*: Stejneger's beaked whale, *L. obli*: Pacific white‐sided dolphin, *P. phoc*: Harbor porpoise, and *P. dall*: Dall's porpoise.

We generated 647 Gb of shotgun metagenomic sequencing data from DNA extracted from the intestinal contents of eight cetacean species. One hundred sixty‐seven MAGs were obtained, each estimated to have a completeness of over 50% and contamination below 10% (Table S1, Figure 3). In addition, 1,104,196 complete ORFs were identified; however, the accumulation curve did not reach a plateau (Figure 3). These observations suggest that microbial diversity may not yet be fully represented. Therefore, caution is required when interpreting the data.

**FIGURE 4:**
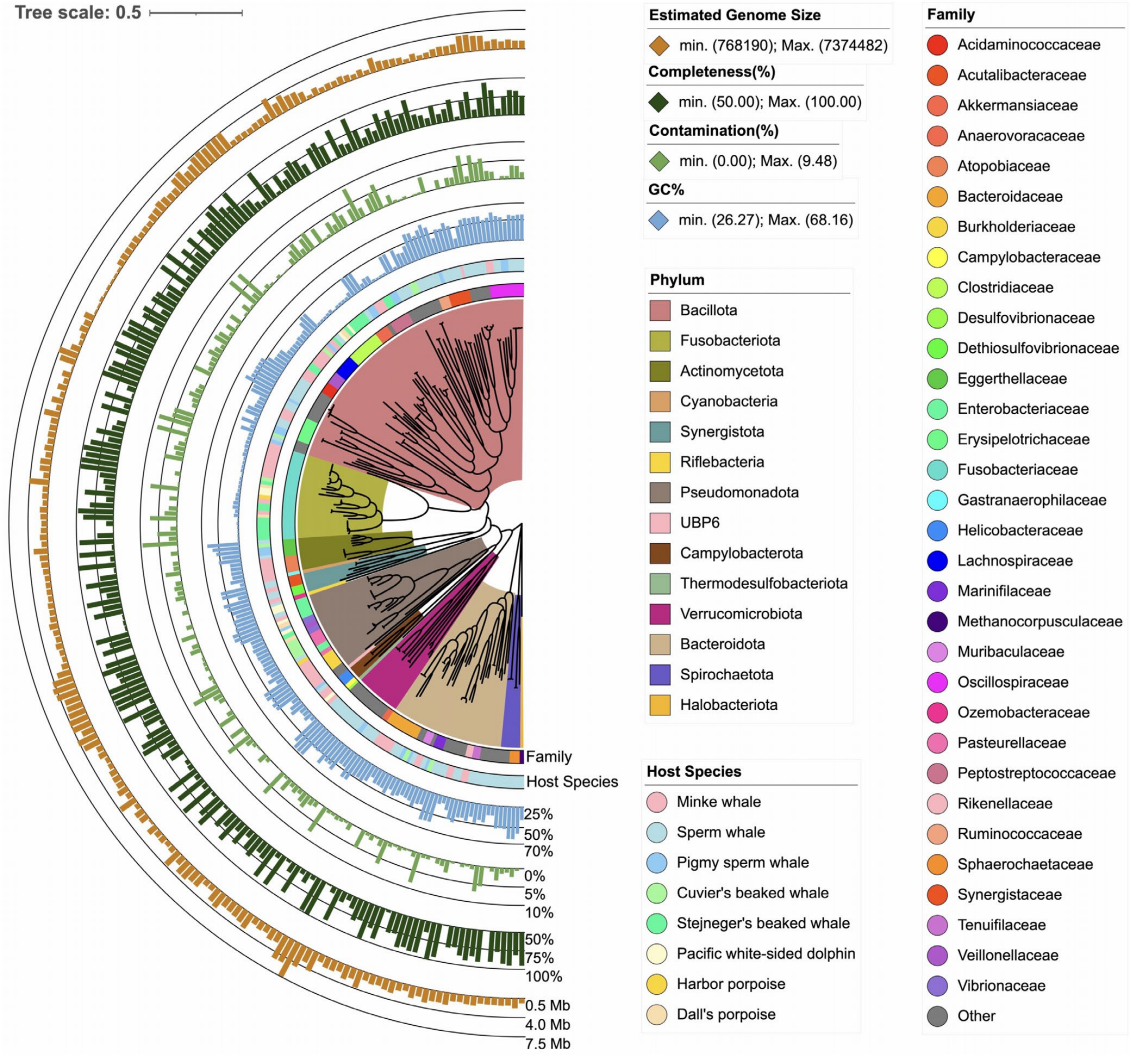
The phylogenetic tree of the reconstructed metagenome‐assembled genomes (MAGs) from the intestinal microbiome of eight cetacean species. The tree depicts the estimated genome size, quality scores, taxonomic classification, and host species for each MAG.

**FIGURE 5:**
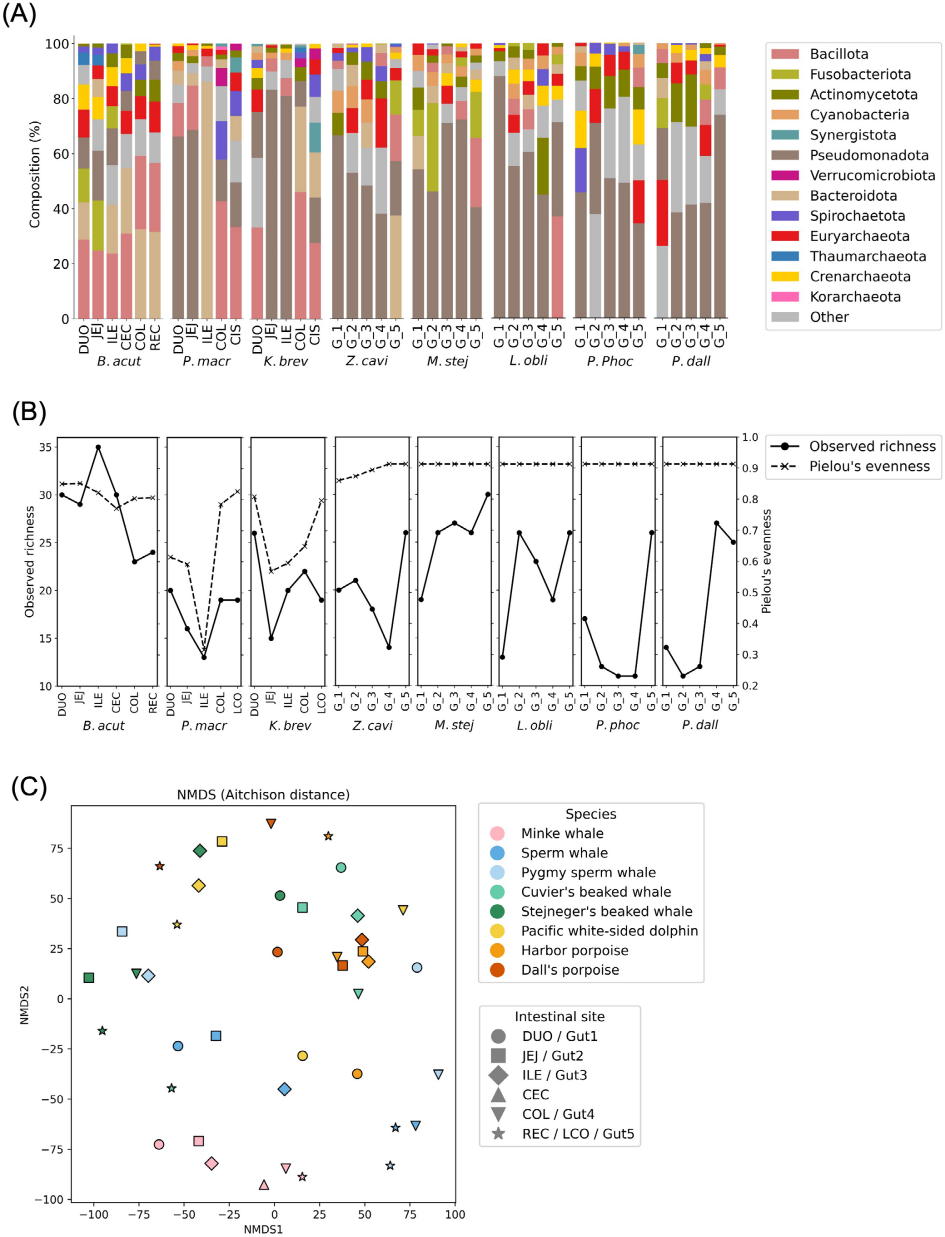
Microbial diversity and community composition in the gut of eight cetacean species. (A) The phylum‐level composition of the gut microbiome across eight cetacean species. Relative abundances are presented as percentages. (B) Alpha diversity indices (observed richness and Pielou's evenness) calculated for each gut region of each species. Values are based on order‐level relative abundance data. (C) NMDS plot based on Aitchison distance, illustrating beta diversity among gut regions and species. NMDS was performed using order‐level relative abundance data. *B. acut*: Minke whale, *P. macr*: Sperm whale, *K. brev*: Pygmy sperm whale, *Z. cavi*: Cuvier's beaked whale, *M. stej*: Stejneger's beaked whale, *L. obli*: Pacific white‐sided dolphin, *P. phoc*: Harbor porpoise, and *P. dall*: Dall's porpoise.

In *K. breviceps* , Bacillota and Bacteroidota were the most abundant phyla in both the colon (COL: 46% and 31%, respectively) and the colonic ampulla (LCO: 28% and 16%, respectively), followed by Pseudomonadota (COL: 9%; LCO: 17%). *P. macrocephalus* exhibited dominance of Bacillota in both COL (43%) and LCO (33%) regions, whereas Pseudomonadota was the second most abundant phylum (COL: 15%; LCO: 16%), with Bacteroidota composing a smaller fraction of the community (Figure 5A, and detailed in the supplemental data). Additionally, a relatively high abundance of the phyla Verrucomicrobiota and Synergistota was observed in the physeteroid colonic microbiome (Figures 4 and 5A). This pattern aligned with previous reports based on 16S amplicon analyses of physeteroid fecal samples (Erwin et al. 2017; Li et al. 2019; Denison et al. 2020).

In contrast, the bacterial communities found in the small intestine—specifically the duodenum and jejunum of *P. macrocephalus* and the jejunum and ileum of *K. breviceps* —were relatively uniform, with Pseudomonadota as the dominant phylum (*P. macr*_DUO: 66%; *P. macr*_JEJ: 69%; *K. brev*_JEJ: 83%; *K. brev*_ILE: 81%) (Figure 5A). The ileum of *P. macrocephalus* exhibited an overwhelming dominance of Bacteroidota (86% of relative abundance), deviating markedly from other gut regions and individuals. Throughout the entire gut, physeteroids showed a notably reduced abundance of the phylum Fusobacteroidota compared with other cetaceans (Figures 3 and 5A).

Among the odontocetes exhibiting a relatively monotonous gut structure ( *Z. cavirostris* , *M. stejnegeri* , *L. obliquidens* , *P. phocoena* , and *P. dalli* ), the phylum Pseudomonadota constituted the predominant group. Conversely, the phylum Bacteroidota was notably scarce (Figure 5A). An exception was identified in the G_5 region of *Z. cavirostris* , where the phylum Bacteroidota emerged as the dominant group. The predominance of Bacteroidota in the G_5 region—located immediately proximal to the anus—aligns with previous findings derived from the 16S amplicon analysis of fecal samples from *Z. cavirostris* (Radaelli et al. 2024). However, this feature was not observed in *M. stejnegeri* , another member of the odontocete family Ziphiidae (Figure 5A).

Despite substantial individual variation, some general trends were observed in α‐diversity metrics across gut regions (Figure 5B). In odontocetes except physeteroids, proximal intestinal samples tended to exhibit lower diversity, while distal regions showed relatively higher richness. In contrast, physeteroids displayed a somewhat distinct pattern: diversity was higher in the duodenum, decreased toward the mid‐intestine (jejunum or/and ileum), and then increased again in the colonic region. Notably, physeteroids showed relatively lower richness and evenness in their colonic samples compared to other species, suggesting a more selective or restricted bacterial community in these regions. In the NMDS plot based on Aitchison distances, samples from *B. acutorostrata* appeared to cluster within species and followed a gradient consistent with gut regional transition (Figure 5C). In contrast, most odontocetes showed greater compositional dispersion and lacked species‐ or region‐specific clustering (Figure 5C). Notably, however, colonic samples (COL and LCO) from physeteroids tended to form a distinct cluster, suggesting potential reduced variability in hindgut microbial communities (Figure 5C).

### 3.2 Metabolic Functions of the Colonic Microbiome in Physeteroids

**FIGURE 6:**
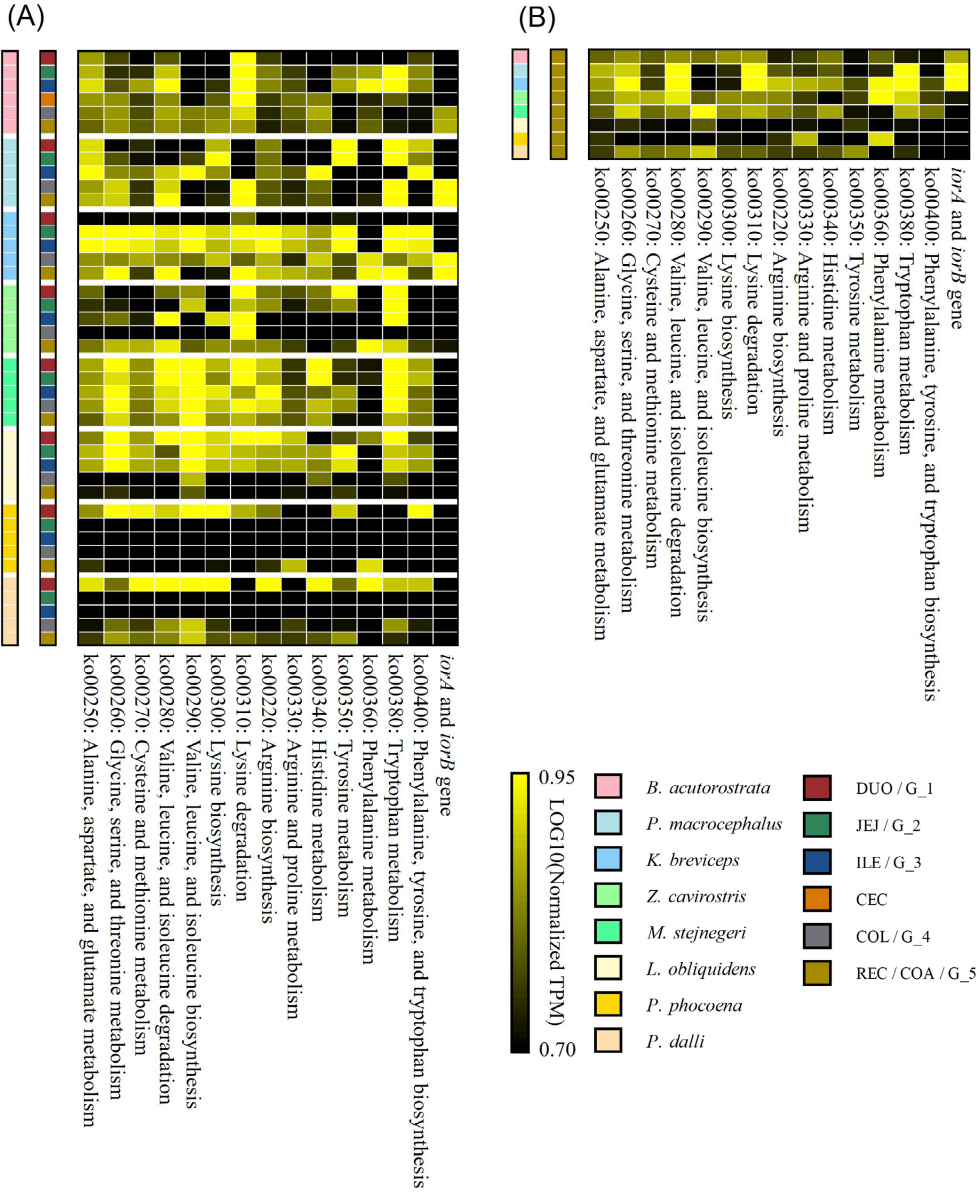
Heatmaps showing the normalized mean TPM values of genes annotated to KEGG amino acid metabolism pathways and indolepyruvate ferredoxin oxidoreductase alpha and beta subunits (*iorA* and *iorB*, respectively) across various intestinal regions and cetacean species. (A) A heatmap of all intestinal regions across all cetacean species. (B) A heatmap of only the distal‐most intestinal regions in each species (rectum, colonic ampulla, or G_5).

**FIGURE 7:**
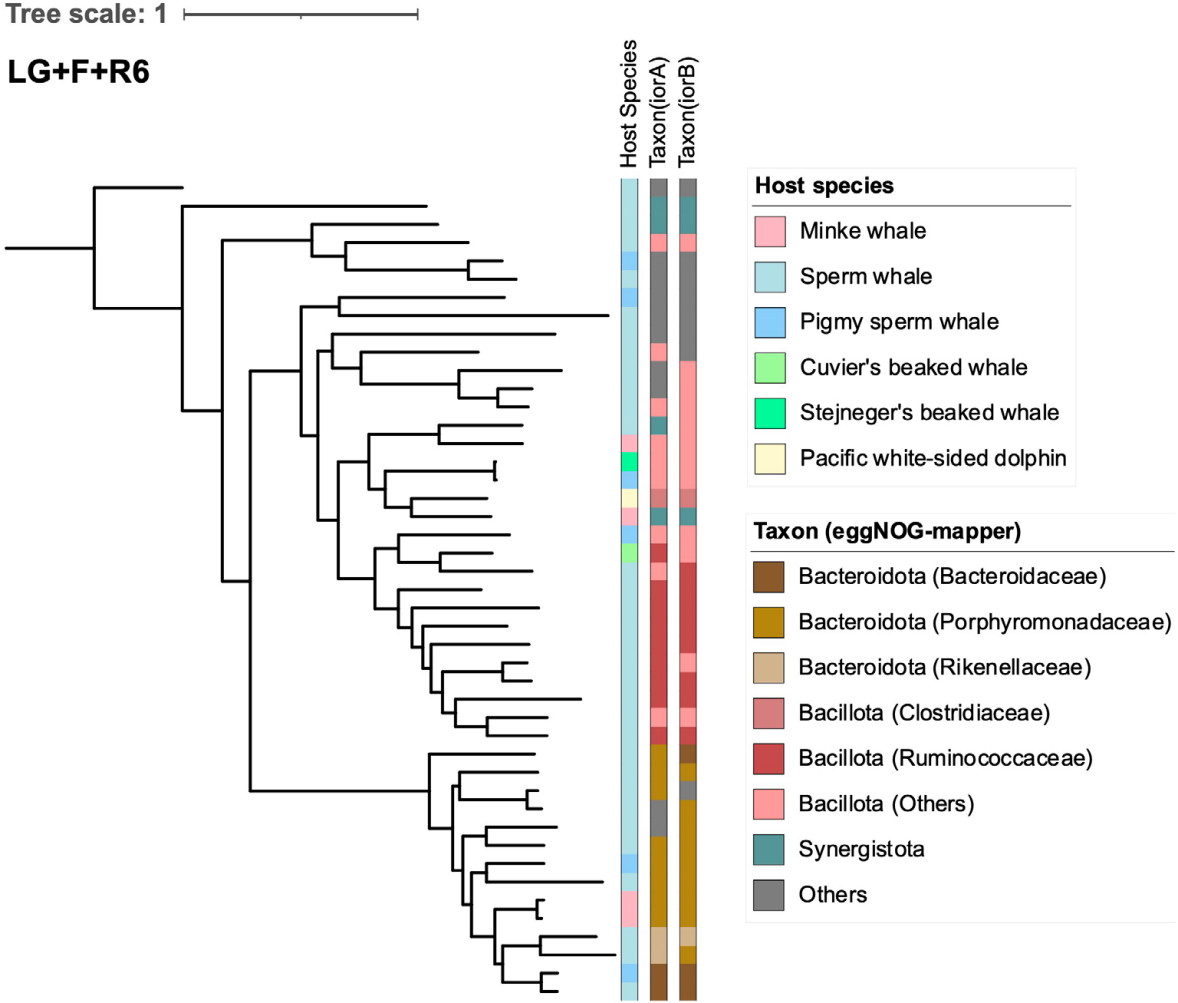
The phylogenetic tree of the indolepyruvate ferredoxin oxidoreductase alpha and beta subunits (*iorA* and *iorB*), which are encoded in tandem. This tree includes the taxonomic classification of the *iorA* and *iorB* sets, along with information on the host species.

For each intestinal region of every cetacean species, we visualized the normalized mean TPM values of the ORFs annotated to various KEGG amino acid metabolism pathways (Figure 6A). In the colonic region of physeteroids, it was observed that genes associated with the degradation of valine, leucine, and isoleucine (ko00280), lysine degradation (ko00310), and tryptophan metabolism (ko00380) were present in higher abundances than those associated with other amino acid metabolic pathways (Figure 6A,B). Among the genes involved in tryptophan metabolism, the indolepyruvate ferredoxin oxidoreductase alpha and beta subunits (*iorA* and *iorB*, respectively), which catalyze the redox reaction of indole‐3‐pyruvate, exhibited a remarkably high relative abundance in the colonic region of the physeteroids (Figures 6A,B and 7).

**FIGURE 8:**
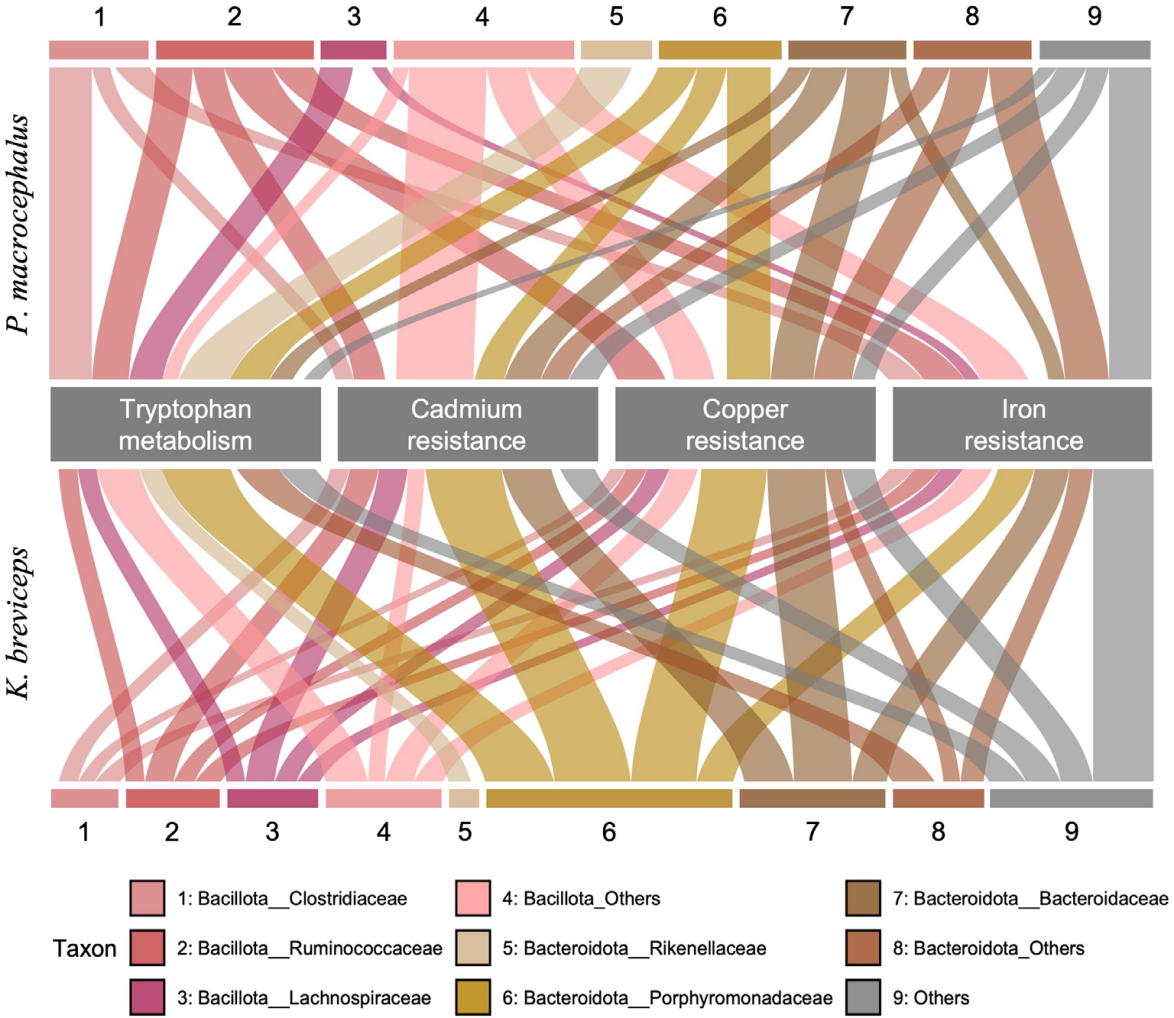
An alluvial plot illustrating the core microbiome in the colonic regions of *Physeter macrocephalus* (top) and *Kogia breviceps* (bottom) encoding genes for tryptophan metabolism (ko00380), cadmium resistance, copper resistance, and iron resistance. The thickness of the alluvium lines represents the mean TPM values observed in the colon and colonic ampulla of *P. macrocephalus* and *K. breviceps* .

The primary bacterial taxa contributing to tryptophan metabolism (ko00380) and heavy‐metal resistance, such as cadmium, copper, and iron, in the colonic region of physeteroids were members of the phylum Bacteroidota, which include the families Rikenellaceae, Porphyromonadaceae, and Bacteroidaceae, in addition to members of the phylum Bacillota, which encompass the families Clostridiaceae, Ruminococcaceae, and Lachnospiraceae. Bacterial members of these families exhibited a relatively high abundance of genes involved in the tryptophan metabolism pathway (Figure 8). In contrast, members of phyla other than Bacillota and Bacteroidota made marginal contributions to this pathway (Figure 8). Similarly, these microbial taxa were also the major contributors to heavy‐metal resistance in the colonic region of physeteroids (Figure 8).

## 4 Discussion

### 4.1 Overview and Significance of the Study

To the best of our knowledge, this study presents the first report investigating the potential involvement of gut symbiotic microbes in the production of tsunabi‐ink through a metagenome catalog of gut microbiomes from eight cetacean species, including *P. macrocephalus* and *K. breviceps* . To date, the characterization of the odontocete gut microbiome composition has been limited to studies using 16S amplicon sequencing. This study provides the first taxonomic composition data based on shotgun metagenomic sequencing. Furthermore, it represents the first study to report shotgun metagenomic data from multiple intestinal regions of cetaceans. Moreover, to our knowledge, the intestinal microbiome of Stejneger's beaked whale ( *M. stejnegeri* ), harbor porpoise ( *P. phocoena* ), and Dall's porpoise ( *P. dalli* ) had not been previously reported even at the 16S amplicon sequence level; thus, our data represent the first record for these species.

Due to the limited sample size (*n* = 1 or 2 per gut region and species), formal statistical comparisons of taxonomic or functional profiles were not feasible. Therefore, all comparative statements in this study should be interpreted as descriptive observations rather than statistically validated differences. While this limitation constrains inferential power, it does not diminish the exploratory value of the metagenomic catalog presented here. While TPM normalization facilitates exploratory comparisons of gene abundance patterns, it does not fully account for differences in genome content, community complexity, or sample size across species. As such, interspecies comparisons in this study are intended to be descriptive rather than quantitative, and caution is advised in interpreting relative abundances across taxa. Notably, the ORF accumulation curve did not reach a clear plateau, suggesting that additional sequencing would likely yield more novel genes and pathways. This incomplete saturation may limit the comprehensiveness of interspecific comparisons and underscores the need for further sampling to fully characterize gut microbial functional repertoires in cetaceans.

Several observations in this study—such as the dominance of Bacteroidota in the distal gut of *Z. cavirostris* , the reduced abundance of Fusobacteria in physeteroids, and the enrichment of amino acid degradation and heavy‐metal resistance pathways—warrant future investigation. While these findings are ecologically and physiologically intriguing, their underlying causes remain unclear. These patterns may reflect interspecific differences in diet, gut morphology, retention time, or host–microbiota interactions, but dedicated mechanistic studies will be required to resolve these hypotheses. We highlight these findings as exploratory leads for future functional and comparative studies.

Although descriptive α‐diversity trends were noted, these patterns should be interpreted cautiously due to limited sample replication and potential biases in DNA recovery. In particular, the proximal gut region of animals is known to contain a high proportion of host DNA, which may reduce the resolution of bacterial community profiling in shotgun metagenomic data. The relatively low richness and evenness in physeteroid colonic regions may reflect ecological filtering, microbial specialization, or technical limitations related to sample composition. Future studies using optimized microbial DNA enrichment protocols will be required to more accurately characterize these regions. Although speculative due to the small sample size, the apparent clustering of colonic microbiomes and relatively lower richness and evenness in the colonic region of physeteroid species may reflect shared colonic environments or functional constraints, potentially associated with their specialized digestion or tunabi‐pigment accumulation processes. Given that tunabi is thought to be formed in the distal colon, this regional microbial similarity could suggest ecological or metabolic constraints favoring specific microbial assemblages. However, further studies with increased sampling depth and functional validation are needed to explore this possibility.

### 4.2 Diet and Colonic Environment of Physeteroids

Odontocetes, including physeteroids, are opportunistic feeders that primarily consume cephalopods, particularly squid (Evans and Hindell 2004; Matsuda et al. 2023). Squid are recognized for their ability to bioaccumulate high concentrations of cadmium (Cd) via trophic transfer (Bustamante et al. 1998, 2002; Calderón et al. 2021) and possess considerable quantities of copper (Cu), which plays a crucial role in their oxygen transport mechanism through the protein hemocyanin (van Holde and Miller 1995; Chignell et al. 1997). Furthermore, physeteroids have evolved a specialized digestive physiology characterized by the development of an enlarged distal colon, in which reddish‐brown feces are temporarily stored.

This unique digestive trait may enable the accumulation of heavy metals from the diet, resulting in prolonged exposure of the gut environment to these elements. Previous studies have reported that the feces of physeteroids contain notably higher concentrations of cadmium, copper, and iron, reaching levels up to a thousand parts per million (Ratnarajah et al. 2014; Page et al. 2024). These concentrations are several to tens of times greater than those observed in other cetaceans, including the blue whale ( *Balaenoptera musculus* ), fin whale ( *Balaenoptera physalus* ), Gervais's beaked whale ( *Mesoplodon europaeus* ), and melon‐headed whale ( *Peponocephala electra* ) (Ratnarajah et al. 2014; Page et al. 2024).

These metals are metabolically relevant to the host and may also influence the composition of the gut microbial community, leading to shifts in its functional traits. Excessive concentrations of copper, cadmium, and iron are harmful to microorganisms because of their toxicity, which can induce oxidative stress and disrupt essential metabolic pathways (Påhlsson 1989; Kumar et al. 2021). In response, many bacteria have evolved various mechanisms to mitigate heavy‐metal toxicity, including the production of pigments that bind to and neutralize heavy‐metal ions. For instance, violacein, an indole‐derived purple pigment, has been demonstrated to confer resistance to cadmium in bacteria (Hui et al. 2022). Similarly, melanin, a phenol‐ and indole‐containing polymer, acts as a potent heavy‐metal chelator (Hayat et al. 2023; Kolipakala et al. 2024).

### 4.3 Indole Compounds Derived From Tryptophan Metabolism and the Tsunabi‐Ink Production

Our study indicates that tsunabi‐ink may be produced as a metabolic byproduct of gut microbial communities responding to heavy‐metal accumulation in the physeteroid gut. *iorA* and *iorB* are enzymes involved in the redox reactions of indole‐3‐pyruvate (Mai and Adams 1994; Porat et al. 2004) and are reportedly associated with pigment production in certain bacteria. Indole‐3‐pyruvate undergoes spontaneous oxidation or enzymatic modifications, resulting in the formation of various pigments, including reddish‐brown compounds (Zuther et al. 2008; Brunke et al. 2010; Vercruysse et al. 2018; Hui et al. 2022). A mutant strain of *Phaeobacter gallaeciensis* WP56 lacking *ior* exhibited reduced pigmentation compared with the wild‐type strain (Berger et al. 2012). *iorA* and *iorB* are potentially associated with pigment production, utilizing indole‐3‐pyruvate as a precursor.

Furthermore, indole‐3‐pyruvate is recognized for its ability to form complexes with iron ions, resulting in brown pigmentation, a characteristic extensively employed in microbiology, particularly in the indole‐3‐pyruvate test utilizing SIM medium (López‐Islas et al. 2024). If a similar complex formation occurs within the colonic ampulla of physeteroids, it could be associated with the pigmentation mechanism of tsunabi‐ink.

The GTDB‐Tk analysis in this study identified numerous bacterial species with experimentally confirmed capabilities for indole compound production among the MAGs derived from the physeteroid gut. These species include *Bacteroides stercoris* (Johnson, Moore, and Moore 1986), *Bacteroides thetaiotaomicron* (Cato and Johnson 1976), *Bilophila wadsworthia* (Claros et al. 1999), *Escherichia coli* (Newton and Snell 1965), *Gabonibacter massiliensis* (Mourembou et al. 2016), *Parabacteroides distasonis* (Ahmed et al. 2019), *Paraclostridium sordellii* (Tamai and Nishida 1964), and *Photobacterium damselae* (Ruixuan et al. 2013). Although *A. muciniphila* does not directly metabolize tryptophan, it has been reported to exhibit a positive correlation with intestinal indole concentrations (Yin et al. 2021).

### 4.4 Pigment Production via Intracellular Iron Uptake

The formation of tsunabi‐pigments may also involve metabolic pathways associated with iron uptake. A high abundance of genes annotated to the order Bacteroidales, including the families Bacteroidaceae, Porphyromonadaceae, and Rikenellaceae, was detected in the colonic regions of physeteroids (Figure 8). Several bacteria of the order Bacteroidales reportedly internalize iron and produce iron‐derived black pigments (Love et al. 1994; Nakayama 2015).

A previous study has shown that iron intake dramatically alters the composition of the gut microbiome in mice; specifically, the order Bacteroidales, including the family Porphyromonadaceae, are susceptible to host iron deficiency and are irreversibly lost from the mouse microbiome (Coe et al. 2021). Because iron is an essential nutrient for many microorganisms (Andrews 1998), specific bacteria may have adapted to excess iron levels by accumulating iron, thereby promoting the production of black pigments. Given the high iron concentrations reported in the feces of physeteroids (Ratnarajah et al. 2014; Page et al. 2024), it is reasonable to conclude that a portion of the black pigment in tsunabi‐ink originates from iron‐utilizing bacteria.

### 4.5 Potential Links Between Amino Acid Catabolism and Tunabi‐Ink Production

Among the metabolic pathways enriched in the colonic region of *P. macrocephalus* , the lysine degradation pathway (ko00310) is of particular interest in the context of tunabi‐ink production. While its direct involvement in pigment biosynthesis remains speculative, recent studies suggest that lysine catabolism may contribute to microbial adaptation under heavy metal stress. For instance, the ectomycorrhizal fungus *Pisolithus albus* exhibits upregulated lysine degradation gene expression when exposed to cadmium and copper (Chot et al. 2023). Similarly, in *Bacillus thuringiensis* and *Citrobacter freundii* , cadmium exposure activates lysine degradation pathways, enhancing urea decomposition and promoting biomineralization (Cai et al. 2025). These processes can lead to precipitation of heavy metals, potentially facilitating their detoxification and sequestration. Furthermore, metal‐chelate formation between lysine and Zn^2+^ or Fe^2+^ has been shown to reduce cadmium uptake in plants such as *Oryza sativa* (Bashir et al. 2018; Ali et al. 2022). These factors could indirectly influence the chemistry of tunabi pigments.

In addition, branched‐chain amino acid (BCAA: valine, leucine, and isoleucine) degradation pathways were also enriched in physeteroid colonic samples. Although their functional significance remains unclear, BCAA metabolism can contribute to microbial energy production and metabolic regulation under anaerobic or stress conditions. Whether these pathways participate in pigment biosynthesis or its regulation in the tunabi‐ink system remains an open question for future targeted investigations.

### 4.6 Symbiotic Relationship Between Physeteroids and Colonic Microbial Communities

Several examples of symbiotic microorganisms contributing to host visual defense mechanisms have been reported in marine animals. For instance, the marine gastropod *Dicathais orbita* stores a tryptophan‐derived reddish‐purple pigment, “Tyrian purple,” in its hypobranchial gland. It has been suggested that indole‐producing *Vibrio* species play a role in this process (Ngangbam et al. 2015). Additionally, symbiosis with bioluminescent bacteria has been widely documented as a visual defense mechanism through counter‐illumination. Examples include the bobtail squids *Euprymna scolopes* and *Vibrio fischeri* (Nyholm and McFall‐Ngai 2021), the pinecone fish *Monocentris japonica* and *Vibrio fischeri* (Ruby and Nealson 1976), and the spotnape ponyfish *Nuchequula nuchalis* and *Photobacterium leiognathi* (Dunlap et al. 2008). In these cases, the host acquires and retains specific bioluminescent bacteria from the marine environment following birth, using their light‐producing capabilities to evade predation.

However, few studies have examined the adaptive significance of pigments or luminescent compounds produced by symbiotic microorganisms in relation to their survival within symbiotic environments. Herein, we propose a novel hypothesis: the pigment components of tsunabi‐ink are byproducts of the heavy‐metal resistance mechanisms exhibited by gut symbiotic bacteria, which were subsequently incorporated into the host's defense system through evolutionary processes.

In this framework, the tsunabi‐ink of physeteroids may represent a distinct case compared with previously acknowledged symbiotic relationships between marine animals and microorganisms. Rather than the host merely acquiring a microbial function for visual defense, the metabolic shift of gut symbiotic bacteria in response to host dietary evolution may be integrated into the host's defensive mechanism. This evolutionary process demonstrates that host–microbe interactions can facilitate the emergence of host‐adaptive traits in novel ways.

A comprehensive understanding of the “tsunabi‐ink” phenomenon requires the integration of molecular, microbial, and ecological approaches. Elucidating the biochemical origin of the pigment remains a key objective. Promising directions include shotgun metagenomic analyses of the gut microbiome from additional cetacean species and individuals, chromatographic and mass spectrometric identification of pigment components, isolation and characterization of pigment‐producing bacteria from physeteroids, and transcriptomic or metabolomic profiling of intestinal tissues. On the other hand, it is equally important to assess the potential ecological function of this behavior. Anecdotal observations suggest that the reddish‐brown fecal discharge may serve to obscure the animal from view, hinting at a possible anti‐predator strategy. This interpretation is consistent with documented predation risks from killer whales and large sharks, particularly in *Kogia* spp. and *P. macrocephalus* . However, the behavior could also represent a non‐adaptive physiological response, involving passive excretion of microbial metabolites accumulated in the distal colon. Disentangling these possibilities will require interdisciplinary studies that connect mechanistic insights with behavioral and ecological data.

## 5 Conclusion

To the best of our knowledge, we have conducted the first shotgun metagenomic analysis of the physeteroid gut microbiome in relation to tsunabi‐ink production. Our findings indicate that the colon of physeteroids is inhabited by distinct microbial communities associated with heavy‐metal resistance and tryptophan metabolism. This suggests a potential role of these bacteria in the pigmentation of tsunabi‐ink. The phenomenon of tsunabi‐ink may serve as an example of host–gut microbe interactions that transcend the realms of digestion and nutrient absorption, indicating that microbial processes can influence the evolution of host behavior. The foraging ecology and evolutionary adaptations of the digestive physiology of physeteroids have modified the gut environment, resulting in excessive heavy‐metal accumulation. The resulting shifts in gut microbial communities may have been assimilated into the host's visual defense mechanisms, such as tsunabi‐ink. These findings provide novel insights into the ecological dynamics of host–microbe relationships.

## Author Contributions

**Takashi Hayakawa:** conceptualization (equal), funding acquisition (lead), investigation (equal), project administration (lead), supervision (lead), writing – original draft (supporting), writing – review and editing (equal). **Takashi Fritz Matsuishi:** funding acquisition (equal), investigation (equal), project administration (equal), resources (lead), supervision (equal), writing – review and editing (equal). **Hayate Takeuchi:** conceptualization (equal), data curation (lead), formal analysis (lead), funding acquisition (equal), investigation (lead), methodology (lead), project administration (equal), resources (equal), validation (lead), visualization (lead), writing – original draft (lead), writing – review and editing (equal).

## Conflicts of Interest

The authors declare no conflicts of interest.

## Supporting information


**Data S1:** The taxonomic information of the reconstructed MAGs of all eight cetacean species.

## Data Availability

Shotgun metagenome data determined in this study were deposited in the DDBJ database with accession numbers PRJDB20379. The taxonomic information of the reconstructed MAGs, the taxonomic and functional annotations of the non‐redundant ORFs, the fasta file of the reconstructed MAGs of all eight cetacean species, and the fasta file of the ORFs catalog of each cetacean species is publicly available in the Figshare repository and can be accessed at https://doi.org/10.6084/m9.figshare.28643699.v1 and https://doi.org/10.6084/m9.figshare.29446625.v1.
